# Optimization of the Filler Concentration on Fused Filament Fabrication 3D Printed Polypropylene with Titanium Dioxide Nanocomposites

**DOI:** 10.3390/ma14113076

**Published:** 2021-06-04

**Authors:** Nectarios Vidakis, Markos Petousis, Emmanouil Velidakis, Lazaros Tzounis, Nikolaos Mountakis, John Kechagias, Sotirios Grammatikos

**Affiliations:** 1 Mechanical Engineering Department, Hellenic Mediterranean University, 71410 Heraklion, Greece; vidakis@hmu.gr (N.V.); mvelidakis@hmu.gr (E.V.); mh90@edu.hmu.gr (N.M.); 2 Department of Materials Science and Engineering, University of Ioannina, 45110 Ioannina, Greece; latzounis@uoi.gr; 3 Design and Manufacturing Laboratory (DML), University of Thessaly, 43100 Karditsa, Greece; jkechag@uth.gr; 4 Laboratory of Advanced and Sustainable Engineering Materials (ASEMlab), Department of Manufacturing & Civil Engineering, NTNU-Norwegian University of Science and Technology, Building B’ Teknologivegen 22, 2815 Gjøvik, Norway

**Keywords:** additive manufacturing (AM), three-dimensional (3D) printing, nanocomposites, polypropylene (PP), titanium dioxide (TiO_2_), tensile test, flexural test, Charpy’s impact test, Vickers microhardness, scanning electron microscopy (SEM)

## Abstract

Polypropylene (PP) is an engineered thermoplastic polymer widely used in various applications. This work aims to enhance the properties of PP with the introduction of titanium dioxide (TiO_2_) nanoparticles (NPs) as nanofillers. Novel nanocomposite filaments were produced at 0.5, 1, 2, and 4 wt.% filler concentrations, following a melt mixing extrusion process. These filaments were then fed to a commercially available fused filament fabrication (FFF) 3D printer for the preparation of specimens, to be assessed for their mechanical, viscoelastic, physicochemical, and fractographic properties, according to international standards. Tensile, flexural, impact, and microhardness tests, as well as dynamic mechanical analysis (DMA), Raman, scanning electron microscopy (SEM), melt flow volume index (MVR), and atomic force microscopy (AFM), were conducted, to fully characterize the filler concentration effect on the 3D printed nanocomposite material properties. The results revealed an improvement in the nanocomposites properties, with the increase of the filler amount, while the microstructural effect and processability of the material was not significantly affected, which is important for the possible industrialization of the reported protocol. This work showed that PP/TiO_2_ can be a novel nanocomposite system in AM applications that the polymer industry can benefit from.

## 1. Introduction

Additive manufacturing (AM) currently has a major role in developing a sustainable economy, with several research projects focusing on sustainability being either AM-based or AM related [1]. Most of the materials used in AM applications are either enhanced polymers, i.e., polymer blends, polymer/small molecule as viscosity modifiers, etc., or polymer-based composite materials [2,3], with the main stock materials being thoroughly studied in the literature [4,5,6]. Research is being conducted regarding the development of polymer-based composite materials, to be employed in advanced applications, using AM. Additive manufacturing, and especially fused filament fabrication (FFF) technology, has great potential in the development of a sustainable society [7]. Relatively, research in AM has been conducted on recycling processes of polymers [8,9,10,11] and on mechanical, thermal, electrical and/or other property enhancements, using a wide variety of fillers and nanofillers dispersed in the polymer matrix [12,13,14,15,16,17].

Among the wide variety of polymers utilized in AM, polypropylene (PP) is of great interest for its high mechanical and thermal stability, making it a proper material for engineering applications, as well as a thermoplastic material that exhibits a great processability via melt-mixing and related compounding processes [18]. PP is widely used by the industry in applications such as operational parts [19] and in the food industry [20]. Specifically, PP is characterized as a high-grade engineering material for AM, but it is only recommended for advanced AM users [21] because it warps easily during the FFF process, and the developed thermal stresses attributed to (i) the 3D printing extruder shear induced crystallization, and (ii) the PP macromolecular chains’ inherent tendency to form crystals on the material coming from the melt to the solid state, resulting in a semi-crystalline polymer [22]. Hence, research has been conducted into the improvement of PP’s printability [17,23] and their mechanical, thermal, and other properties [17]. It could be easily realized that polypropylene has a high potential in FFF AM applications; therefore, it was chosen herein as the polymer matrix material for the development of nanocomposites with enhanced 3D printed specimens’ properties.

Titanium dioxide (TiO_2_) was selected for the current study as the nanofiller for the preparation of nanocomposite materials, as its spherical shape, surface chemistry, and ceramic nature have been proven in the literature to enhance the properties of various polymers [13,16,24]. For instance, titanium dioxide has been investigated, e.g., (i) as nanofiller in order to improve the UV stability of polymers, because of its wide bang gap semiconductor properties and its exceptional UV absorption properties [25], (ii) as a pigment for whitening [26], and (iii) to improve and endow antibacterial properties of polymers and/or other nanoparticles [27]. Research has also been conducted with polymers and TiO_2_ as a filler, focusing on additive manufacturing FFF technology [28,29,30], but no similar research has yet been presented in the literature focusing on PP/TiO_2_ nanocomposites that have been 3D printed with the FFF AM process and fully characterized. Nanocomposites were prepared in various concentrations in order to investigate the effect of the nanofiller in the polymer matrix properties and to specifically highlight the process–structure–property relationship.

In this study, nanocomposite materials were developed with a thermomechanical melt mixing process, using polypropylene (PP) and titanium dioxide (TiO_2_) at various filler loadings. The produced nanocomposite filaments were utilized in the AM FFF process for the manufacturing of specimens suitable for testing, according to international standards. The TiO_2_ filler effect on the PP polymer’s mechanical, viscoelastic, flow, physicochemical, and fractographic properties has been thoroughly investigated. Low filler concentrations were selected to determine the sensitivity of the polymer matrix to this specific nanofiller loading. According to authors’ knowledge and a meticulous literature survey, no similar research has been conducted so far. Detailed mechanical testing, i.e., tensile, flexural, impact, microhardness, and dynamic mechanical analysis (DMA), demonstrated the effect of the nanofiller on the mechanical properties of the polymer matrix. Scanning electron microscopy (SEM) was employed to analyze the microstructure of the fractured surfaces after tensile tests as well as the side surfaces of the 3D printed nanocomposites. Atomic force microscopy (AFM) analysis showed that the extruded filaments’ surface roughness increased in general with the increase of the TiO_2_ filler loading. Raman spectroscopy proved the nanocomposite specific responses, while melt flow volume index (MVR) analysis revealed the effect of the nanofiller on the flow properties of the polymer matrix. It was found that, overall, the properties of the polymer matrix improved with the increase in the filler concentration, up to the maximum concentration studied in this work, while the filler had no significant effect on the printability of PP. The nanocomposites in this study were prepared with a versatile, scalable, and industrial ready process, suitable for advanced engineering applications. Additionally, the mechanisms and the effect of the filler on the matrix material were thoroughly investigated, analyzed, and presented so as to ensure the reliability of the results.

## 2. Materials and Methods

### 2.1. Materials

Polypropylene (PP) in a powder form was procured from Hellenic Petroleum S.A. (Athens, Greece), and was used throughout this work, under the trademark of Ecolen PP. According to the supplier technical specification data sheet, the PP was an isotactic homopolymer thermoplastic material. Titanium dioxide (TiO_2_) was procured from Degussa Evonik P25 (Essen, Germany) in the form of nanoscale particles (NPs), with a mean diameter in the range of 25–50 nm. In order to investigate the effect of the titanium dioxide nanofiller in the polypropylene matrix, nanocomposite materials were fabricated at four (4) different filler’s concentrations, i.e., 0.5 wt.%, 1 wt.%, 2 wt.%, and 4 wt.%. The pure PP’s properties were also studied and evaluated with respect to the PP/TiO_2_ nanocomposites prepared at the different concentrations.

### 2.2. Methods

**Figure 1:**
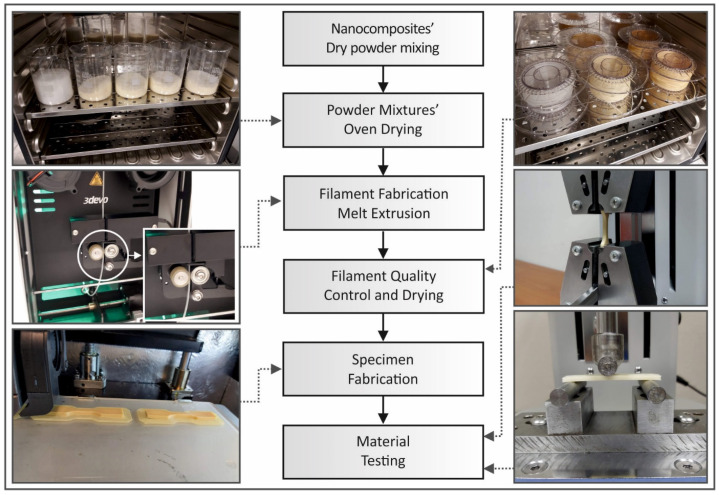
Flow chart of the methodology steps followed in this study and the representative pictures captured during the individual processes.

The methodology and a brief presentation of each step followed in this study are shown in Figure 1. The methodology is described in detail in the following sections.

#### 2.2.1. Filament Fabrication

Pure PP and all of the nanocomposite’s concentrations in this work were fabricated under same conditions, initially in filament form. Each material was weighted and mixed, with the filler’s percentage calculated per weight (wt.%). The mechanical mixing procedure was conducted in a closed chamber to reduce the loss of the TiO_2_ filler, because of its high hovering behavior. Before starting the mixing process, PP and TiO_2_ were dried for 24 h at 80 °C in a laboratory oven. After homogenization, each mixture was further dried for 5 h at 80 °C in the same oven before proceeding to the filament extrusion step.

In order to produce filaments as feedstock for the AM process in this study, a 3D Evo’s (3D Evo B.V., NL) Composer 450 single screw extruder was employed. In this extruder, a total of four (4) heating zones were used for the polymer’s melting and extrusion. The rotational speed of the screws could be adjusted by the user and an adjustable built-in winder regulated the winding speed with feedback from the built-in filament’s diameter measurement sensor. A real time, during the extrusion process, filament diameter measurement diagram provided quality control measurements to evaluate whether the 1.75 mm filament diameter required by the 3D printers was retained.

All of the materials were extruded under the same conditions (pure PP and four nanocomposites), and a mean deviation of 0.08 mm was achieved for the filaments’ diameters throughout the process. The extrusion temperature settings were 195 °C at heat zone 4 (closer to the hopper), 210 °C at heat zone 3 and heat zone 2 (melt-mixing stage), and 205 °C at heat zone 1 (closer to the extruder’s nozzle). Extruder’s screw rotational speed was adjusted to 3.5 rpm and fans (built-in) utilized for cooling were adjusted to 40%. A special duct was designed and manufactured (through AM) for the purposes of this study. This duct was designed to direct air from the extruder fans to the extruded material in the 3D Evo’s nozzle, so as to improve the air flow around this area. This was deemed necessary for these specific materials of the study. In this way, a smoother cooling process was achieved for the extruded material, and the filament showed a higher roundness, leading to an improved 3D printing quality. Filament’s diameter and roundness were further measured manually with a high-quality caliper prior to the 3D printing process, as an additional measure, in order to ensure the quality of the extruded filament.

#### 2.2.2. Tensile Specimens’ Fabrication and Testing

**Figure 2:**
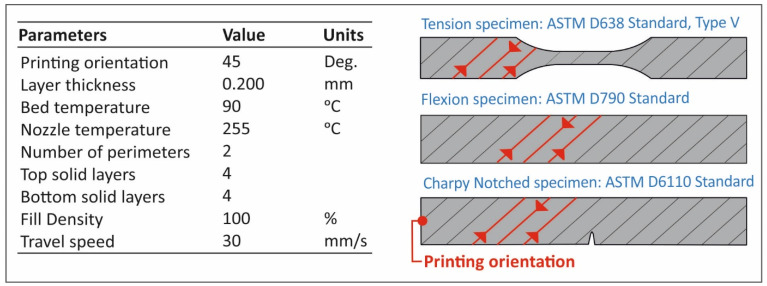
Fused filament fabrication (FFF) additive manufacturing (AM) settings used in the 3D printing process for all of the specimens manufactured in this study.

Fused filament fabrication (FFF) additive manufacturing (AM) technology was chosen for the specimens’ fabrication. Intamsys Funmat HT 3D printer (Intamsys Technology Co. Ltd., Shangai, China) was used with a 0.4 mm nozzle setup. In Figure 2, the 3D printing process settings are shown. Additionally, the nozzle’s fans were closed during the fabrication procedure. Intamsys Funmat HT is a total-closed chamber 3D printer able to achieve a thermal stability in the 3D printing process, which is crucial for PP’s 3D printing, without intense warping problems. The settings of the FFF process not shown in Figure 2 were set to default, according to the Intamsuite slicer software used for the purposes of the current study. The default values were set by choosing PP as the material from Intamsuite’s materials list. The same parameters were used in all of the specimens manufactured in the study.

Tensile tests were conducted according to the American Society for Testing and the Materials (ASTM) D638-02a international standard. Five type V specimens with a 3.2 mm thickness were fabricated for each material, according to the standard. An Imada MX2 (Imada Inc., Northbrook, IL, USA) apparatus was used for the tensile tests. The elongation speed was adjusted to 10 mm/min and all of the tests were conducted under room temperature conditions (22 °C, ~50% RH).

#### 2.2.3. Flexure Specimens Fabrication and Testing

The same procedure and settings were followed for the flexural test specimens’ fabrication. ASTM D790-10 was the international standard followed for the flexural tests. Five specimens were fabricated, with a thickness of 3.2 mm, for each material tested. A total of 25 specimens were tested in a three-point bending setup in the same device described above (Section 2.2.2), according to the standard. The bending speed was set to 10 mm/min, while room temperature conditions prevailed during testing (22 °C, ~50% RH).

#### 2.2.4. Charpy’s Impact Specimens Fabrication and Testing

The impact test of Charpy’s notched process was conducted for the purposes of the current study. The ASTM D6110-04 standard was followed, while the specimens’ dimensions were 80 mm length, 10 mm width, and 8 mm height. Specimens were fabricated with the exact same parameters described above (Section 2.2.2). A Terco MT220 Charpy (Terco AB, Kungens Kurva, Sweden) impact test machine was used in the current study. Hammer’s release height was the same for all of the tests conducted (367 mm). Charpy’s impact notched tests were carried out under room temperature conditions (22 °C, ~50% RH).

#### 2.2.5. Micro-Hardness Measurements

Microhardness is a very important material property, related to the plasticity of the material, which is directly connected with the material mechanical response [31]. Vickers microhardness measurements were also conducted for all of the materials fabricated in this study. The ASTM E384-17 standard was followed for the testing procedures. Measurements were conducted on randomly selected areas in the tensile, flexure, and impact specimens. The specimen surfaces were fully polished prior to each set of measurements. Tests were carried out on an Innova Test 300-Vickers machine (Innovatest Europe BV, Maastricht, The Netherlands). The applied force for indentations was set to 300 gF, while the duration for the indentation was set to 10 s. Five measurements of imprints were conducted for each nanocomposite material and pure PP. The indentations were carried out at room temperature conditions (22 °C, ~50% RH).

#### 2.2.6. Dynamic Mechanical Analysis (DMA)

Dynamic mechanical analysis (DMA) was conducted on the specimens of all of the materials fabricated for this study. The apparatus used for this test was a TA Instruments DMA850 instrument (TA Instruments, New Castle, DE, USA).

Prior to testing, the samples were dried at a temperature of 30 °C for a minimum of 48 h. Because of the samples having rough side edges from the manufacturing process, the samples were polished in two steps using 240 and 400 grain sandpaper under water flow.

The DMA testing procedure consisted of a temperature ramp from room temperature to 130 °C (and in some cases up to 135 °C), at a rate of 3 °C min^−1^. Testing was conducted using the three-point bending fixture. The samples were preloaded to 0.1 N. A sinusoidal displacement was applied to the samples with a constant amplitude of 30 µm and a frequency of 1 Hz throughout the tests. The data were collected by the instrument at a sampling rate of 0.33 Hz. The recorded parameters were the storage modulus, loss modulus, tan δ, temperature, time, and oscillation angular frequency.

#### 2.2.7. Characterization Techniques

Raman spectroscopic analysis was carried out for the pure 3D printed PP, as well as the PP/TiO_2_ nanocomposite specimens acquiring spectra from the top 3D printed layer surface. All of the spectra were obtained using a Labram HR-Horiba (Kyoto, Japan) scientific micro-Raman system, while all of the spectra were treated with a baseline correction through the subtraction of a linear or polynomial fit of the baseline from the raw spectra, in order to remove the tilted baseline caused by various noises, i.e., fluorescent background, etc. For all of the Raman experiments, an optical microscope equipped with a 50× long working distance objective was used for delivering both the excitation light, as well as for collecting the back-scattering Raman activity. An Ar^+^ ion laser line at a 514.5 nm wavelength with 1.5 mW power at the focal plane was utilized for the Raman excitation.

Scanning electron microscopy (SEM) microstructural investigations were performed using a JEOL JSM 6362LV (Jeol Ltd., Peabody, MA, USA) electron microscope in high-vacuum mode at 20 kV acceleration voltage on sputtered-gold coated samples. The images from the side surface and the fracture area of the randomly selected tensile test samples from all of the filler loadings tested in this study were captured at various magnifications.

The filaments’ surface topology was studied using atomic force microscopy (AFM) in tapping mode (TM-AFM). The AFM images (height data) were recorded with a scanning probe microscope (MicroscopeSolver P47H Pro, NT-MDT, Moscow, Russia) in air at room atmosphere and a temperature of 22 °C, at a resonant frequency of about 300 kHz. Commercially available silicon cantilevers were used with a scanning frequency of 1 Hz, a tip cone angle of 20°, a cantilever spring constant of 35 N/m, and a tip radius of about 10 nm.

The flow properties of the PP and PP/TiO_2_ nanocomposites were tested using an Instron CEAST MF20 Melt Flow Tester (Instron Corp., Norwood, MA, USA). The tests were conducted following ASTM D1238-10, on all of the samples, employing dead weights of 2.16 kg.

## 3. Results and Discussion

### 3.1. Mechanical Properties Results

#### 3.1.1. Tensile Test Results

**Figure 3:**
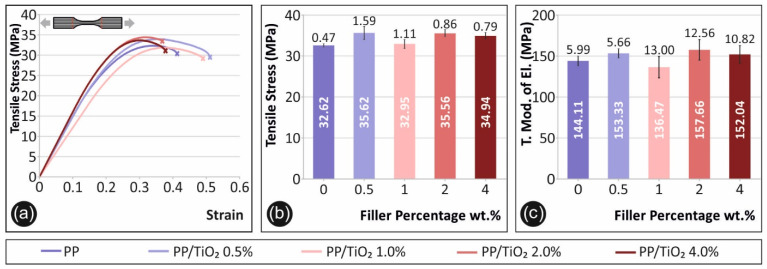
Tensile test results: (**a**) representative specimen’s tensile stress–strain graphs, (**b**) average maximum tensile stress values calculated (MPa) and the respective deviations in comparison with the filler’s concentration for each fabricated material, and the (**c**) average tensile modulus of elasticity (MPa) and the respective deviations in comparison with the filler’s concentration for each fabricated material.

In Figure 3, the results from the tensile tests conducted on the specimens of all of the materials produced for the current study are depicted. In Figure 3a, a representative stress–strain graph of each sample case is shown. The average tensile stress values at break (MPa) are presented in Figure 3b, while in Figure 3c, the calculated average tensile modulus of elasticity (MPa) is shown with respect to the filler’s percentage for all of the materials tested in this study.

As shown in Figure 3b, regarding the maximum tensile stress developed during the tensile test, titanium dioxide increased by approximately 10% of the tensile strength of the pure PP at a filler’s loading of 0.5 wt.%, and overall, all of the filler’s concentrations tested in this work improved the tensile strength of pure PP. The filler’s percentage also revealed an effect on the ductility of the materials tested (Figure 3a). Low concentrations of 0.5 wt.% and 1 wt.% revealed a more intense ductile behavior after the yield point, while TiO_2_ concentrations of 2 wt.% and 4 wt.% shifted the material behaviors to being more brittle. Interestingly, both the tensile strength and stiffness exhibited an increase for 0.5 wt.%, then a drop for 1 wt.%, which subsequently changed again exhibiting higher strength and stiffness values for 2 wt.% and 4 wt.%, respectively. The tensile strength and modulus of the elasticity results of this study are in agreement with the results from the PP composites presented in the literature [17]. A similar behavior of using TiO_2_ nanoparticles as a filler to enhance the tensile properties of the polymer matrices is also reported in the literature for two different polymers and similar filler concentrations [1,16].

#### 3.1.2. Flexural Test Results

**Figure 4:**
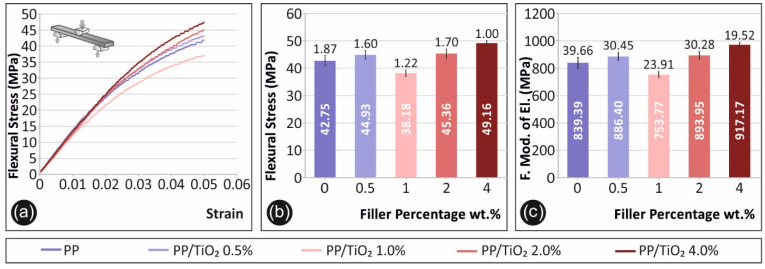
Flexural test results: (**a**) representative specimen’s flexural stress (MPa) to strain graphs, (**b**) average maximum flexural stress values calculated (MPa) and the respective deviations in comparison with the filler’s concentration for each fabricated material, and the (**c**) average flexural modulus of elasticity (MPa) and the respective deviations in comparison with the filler’s concentration for each fabricated material.

In Figure 4, the results from the flexural tests conducted are presented. Figure 4a shows a representative stress (MPa) to strain graph for each material tested in this study. A maximum strain of 0.05 (5%) is shown for all of the materials tested, according to the ASTM D790-10 standard instructions, as no break occurred at the specimens. In Figure 4b, the average maximum flexural stress (MPa) calculated at 5% strain is shown with respect to the material’s filler percentage, while Figure 4c presents the corresponding average calculated flexural modulus of elasticity (MPa) values.

As Figure 4b depicts, the highest value for the flexural stress at a 5% strain was observed for 4 wt.% TiO_2_. The calculated value at this concentration is 15% higher than the pure PP. It should be mentioned that the flexural test was terminated at the 5% strain, according to the ASTM D790-10, and no break occurred for any of the specimens. A similar trend to tensile testing was observed for the flexure testing, which revealed a generally increasing response to nanofiller loading, apart from the case of 1 wt.% TiO_2_.

#### 3.1.3. Impact and Microhardness Test Results

**Figure 5:**
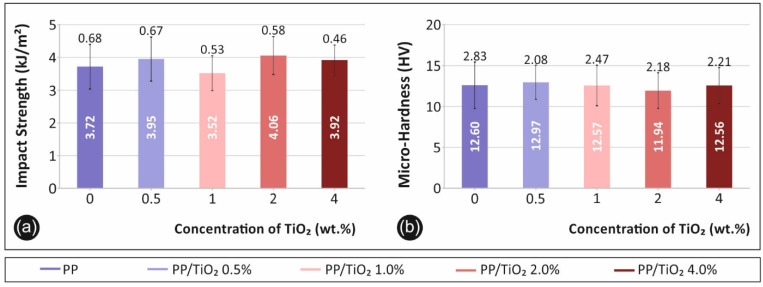
(**a**) The average impact strength (kJ/m^2^) in comparison with the filler’s concentration for each fabricated material and the (**b**) average microhardness (HV) in comparison with the filler’s concentration for each fabricated material.

Charpy’s notched impact test result are shown in Figure 5a. The impact strength (kJ/m^2^) is presented for each material fabricated in the study. Figure 5b presents the corresponding microhardness (HV) calculated values from the imprint’s measurements.

The impact strength (Figure 5a) of the polymer was found to not be significantly affected by the filler’s concentration, although a similar trend to the other tests was observed. This agrees with the literature findings [8,24,32], which report that fillers have a low effect on the impact strength of polymers. A similar trend was observed for the microhardness measurements, with respect to the filler’s ratio (Figure 5b). PP is a well-known ductile material, so a low TiO_2_ filler loading is not expected to affect the PP’s surface microhardness.

### 3.2. DMA Results

**Figure 6:**
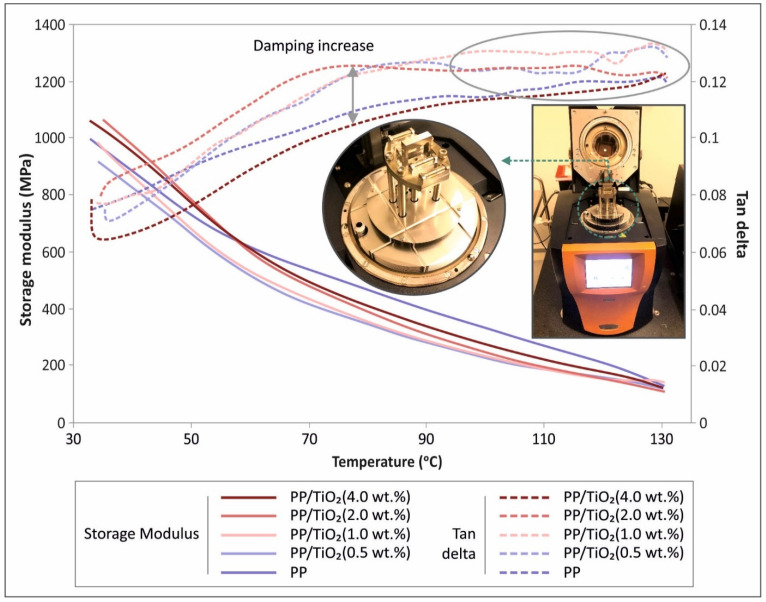
Storage modulus (MPa) left Y axis and tan(d) right Y axis in comparison with the dynamic mechanical analysis (DMA) temperature range for all of the fabricated materials.

In Figure 6, all of the measured data retrieved through DMA are shown. The storage modulus (MPA) curve (left Y axis) to temperature range of test (X axis) is presented for all of the materials tested during the current study. In the same figure (Figure 6) and at the right Y axis, tan(d) is shown for all of the materials as a function of temperature (X axis).

In the DMA thermomechanical analyses, the effects of the filler type and concentration on the storage modulus and tan(d) values, have been examined. In general, storage modulus values at very low temperature levels (~30 °C) are indicative of the flexural modulus of the material, while tan(d) corresponds to damping.

The storage modulus reveals a decreasing tendency, at a high rate, which changes before 70 °C. On the other hand, tan(d) exhibits an oscillation in the data, which becomes prominently significant at high temperature ranges (>100 °C).

In the case of titanium dioxide, the storage moduli generally increased with higher filler fractions, at low temperatures, which confirms the response of the material to flexure (Figure 4). The 0.5 wt.% filler fraction case revealed a lower storage modulus than the reference case values, while the converse was true for the 1, 2, and 4 wt.% groups.

With regards to tan(d), the filler loading generally increased the damping of the investigated nanocomposites, apart from the case of 4 wt.%. The trend of the tan(d) curve was found to be similar for all of the samples, i.e., it increased with a steady rate that became asymptotic after 70 °C, especially for the cases of 0.5, 1, and 2 wt.%. Furthermore, oscillations recorded at higher temperatures are indicative of softening. This effect was not pronounced in the case of 4 wt.% because of the brittleness of the material, as was demonstrated by the tensile and flexural tests. In addition, this effect might be indicative of potential filler agglomeration at high loadings.

### 3.3. Raman Results

**Figure 7:**
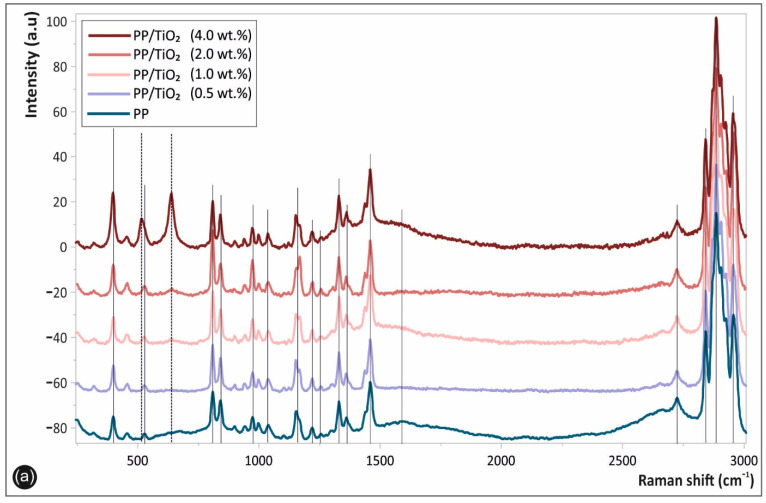

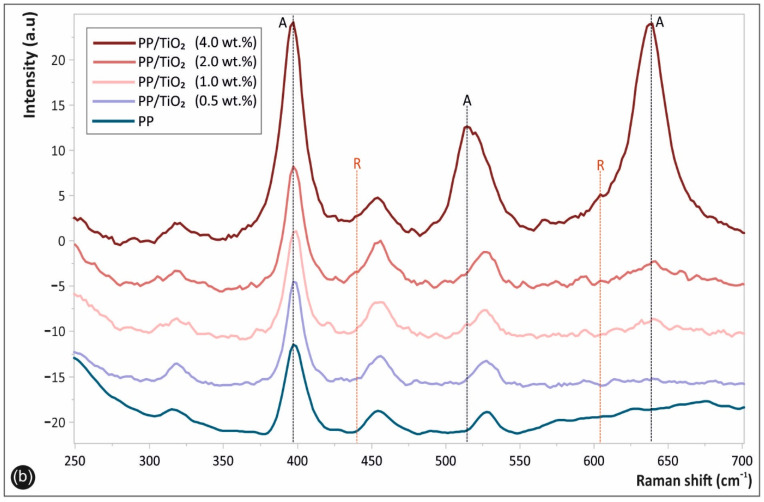
Raman spectra of the 3D printed samples, namely the pure polypropylene (PP) and the PP/TiO_2_ nanocomposites at different filler loadings, in the spectral region of (**a**) 250–3000 cm^−1^ and (**b**) 300–700 cm^−1^, respectively.

Figure 7 shows the Raman spectra of the corresponding 3D printed samples, namely the pure PP, as well as the PP/TiO_2_ nanocomposites at different filler loadings. Specifically, Figure 7a shows the whole acquired Raman spectrum in the spectral region of 250–3000 cm^−1^, while Figure 7b shows the whole acquired Raman spectrum in the spectral region of 300–700 cm^−1^. All of the peaks attributed to the PP matrix macromolecular chains’ chemistry, i.e., the polymer chain backbone and the side groups, are depicted with continuous lines, while the specific bands assigned to the TiO_2_ NPs are illustrated with dashed lines in Figure 7a,b, respectively.

The characteristic PP Raman peaks could be clearly seen at different band positions as being in good agreement with other reported PP spectra in the literature [4]. More specifically, the characteristic fingerprints of PP are located at ca. 385, 810, 868, 967, 1036, 1168, and 1221 cm^−1^ (C–C stretching vibration); 1250 and 1320 cm^−1^ (CH deformation vibration); 1334 and 1454 cm^−1^ (–CH_2_ of the PP backbone macromolecular chains); 1361–1385 cm^−1^ (–CH_3_ deformation vibrations of PP chains, as well as the –CH_3_ side group rocking vibration) [33]; and 2721, 2837, 2875, and 2962 cm^−1^ (CH_3_ symmetric and asymmetric stretching vibration) [34]. The spectra of the PP/TiO_2_ nanocomposites at the different TiO_2_ wt.% filler loadings exhibited some additional peaks attributed to the TiO_2_ nano crystallites’ vibrational modes, indicated more clearly in Figure 7b. Namely, the peaks at ca. 513 cm^−1^ and 639 cm^−1^, as well as the enhanced peak intensity of the band at ~400 cm^−1^, which increased for all of the nanocomposites compared with the pure PP peak intensity, are all attributed to the characteristic TiO_2_ anatase (A) phase vibrational modes (shown with black dashed lines), as reported elsewhere [16].

It is known that anatase (A) and rutile (R) are the most common crystalline phases of TiO_2_, which exhibit characteristic and different Raman active modes [13]. The anatase main peaks are located at 395 (*B*_1g_), 513 (*A*_1g_), and 639 cm^−1^ (E_g_), while the rutile peaks are located at 442 and 605 cm^−1^, respectively [35]. It is worth mentioning that the TiO_2_ NPs utilized herein as the nanocomposite reinforcement fillers consist mainly of the anatase phase; however, a small fraction of rutile phase (R) was detected, due to the peaks appearing at ca. 442 and 605 cm^−1^, more precisely shown in Figure 7b (red dashed lines). Moreover, it could be observed, and it is worth mentioning, that the characteristic Raman peak intensities corresponding to the blended TiO_2_ NPs in the PP matrix increased with the increased filler loading, shown more clearly in Figure 7b.

### 3.4. Microstructural Analysis

#### 3.4.1. SEM Analysis

**Figure 8:**
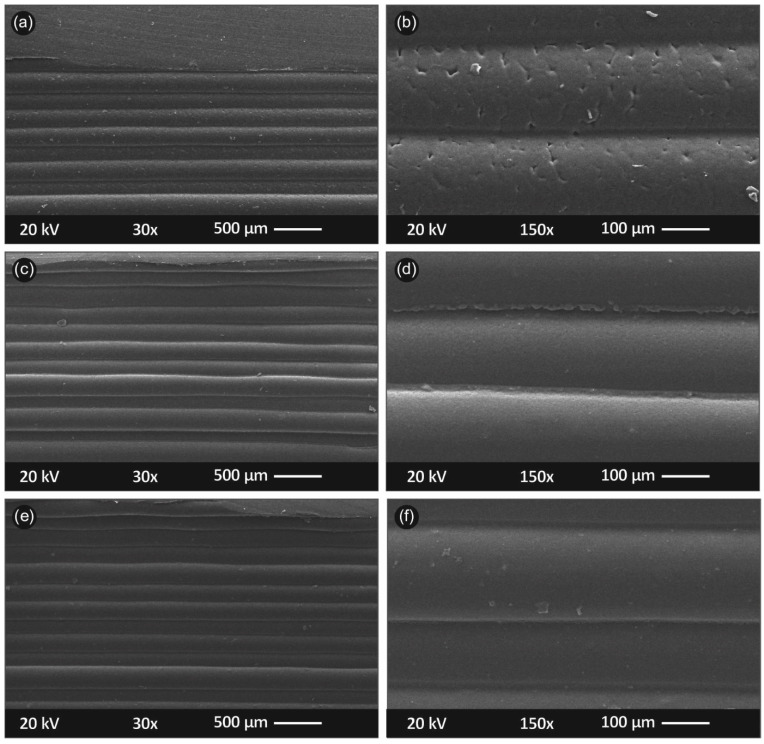
SEM images of the specimens’ side surfaces: (**a**) ×30 magnification of PP/TiO_2_ 0.5 wt.%; (**b**) ×150 magnification of PP/TiO_2_ 0.5 wt.%; (**c**) ×30 magnification of PP/TiO_2_ 2 wt.%; (**d**) ×150 magnification of PP/TiO_2_ 2 wt.%; (**e**) ×30 magnification of PP/TiO_2_ 4 wt.%; and (**f**) ×150 magnification of PP/TiO_2_ 4 wt.%.

In Figure 8, the side surface of the randomly selected tensile test specimens is shown in different magnifications as a means to quantitatively evaluate the samples’ interlayer fusion, interlayer defects and inhomogenities, etc. Specifically, in Figure 8a,b, the side surface images in magnification of ×30 (a) and ×150 (b) are shown for PP/TiO_2_ 0.5 wt.%., respectively; Figure 8c,d shows the side surface of PP/TiO_2_ 2 wt.%, respectively; and Figure 8e,f shows the side surface of PP/TiO_2_ 4 wt.%, respectively.

Figure 8 shows that the nanocomposite materials were 3D printed in an appropriate and optimum 3D printing protocol. The layer deposition was of a fine quality as the layers did not exhibit a high variation in their positioning. From the high magnification images (Figure 8b,d,e), a well interlayer fusion is observed, without any observed voids, discontinuities, and inhomogenities. Some of the tiny defects shown in Figure 8b can likely be attributed to some local malfunction in the extrusion process during 3D printing (probably a tiny remnant inside nozzle), while PP/TiO_2_ 0.5 wt.% performed mechanically soundly, as presented earlier.

**Figure 9:**
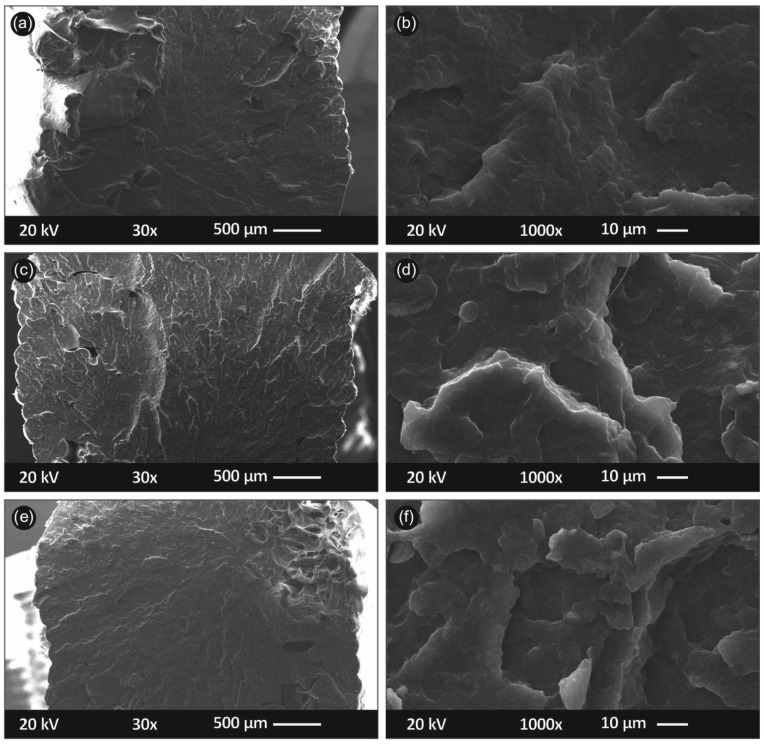
SEM captures of specimens’ fracture area as: (**a**) ×30 magnification of PP/TiO_2_ 0.5 wt.% (**b**) ×1000 magnification of PP/TiO_2_ 0.5 wt.% (**c**) ×30 magnification of PP/TiO_2_ 2 wt.% (**d**) ×1000 magnification of PP/TiO_2_ 2 wt.% (**e**) ×30 magnification of PP/TiO_2_ 4 wt.% (**f**) ×1000 magnification of PP/TiO_2_ 4 wt.%.

Figure 9 shows the PP/TiO_2_ 3D printed nanocomposite specimens’ fractured surfaces (acquired from the representative tensile test fractured samples). In Figure 9a,b, the PP/TiO_2_ 0.5 wt.% specimen’s fractured surface is shown at two different magnifications, of ×30 (Figure 9a) and ×1000 (Figure 9b). Figure 9c,d depicts PP/TiO_2_ 2 wt.% at the same magnifications, while Figure 9e,f depict PP/TiO_2_ 4 wt.% at the same magnifications, respectively.

It can be seen in Figure 9 that the 3D printing process was of an exceptional quality, as the specimens seemed to present no visible porosity because of the inherent nature of the FFF 3D printing filamentous deposition AM process. In this way, the anisotropic effects that are usually observed in 3D printing were minimized. In the images, a somehow ductile fracture area can be observed in Figure 9a, which corresponds to 0.5 wt.%, as opposed to the other cases that correspond to a more brittle failure that agrees with the measured tensile tests results (only in Figure 9a,b, some filaments were found to be pulled out from the fractured surface, indicating some ductile fracture mechanism, unlikely for the other samples). No micro-aggregates can be observed and/or are visible in the 3D printed specimens’ fractured surfaces, even up to the highest nanocomposite’s concentration, i.e., at 4 wt.% (Figure 9e,f). Finally, for all of the nanocomposites processed through the filament extrusion and consecutive FFF 3D printing AM process, overall, (i) a rather good dispersion of nanoparticles was observed (not aggregating phenomena occurred for instance during the melt mixing/processing steps) and a (ii) low porosity between the adjacent filaments with a good interface between the filaments was achieved.

#### 3.4.2. AFM Analysis

**Figure 10:**
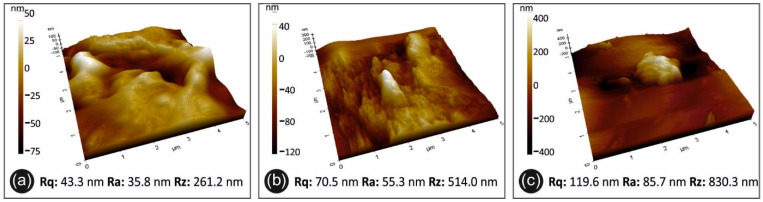
AFM images of the filament surface for the different materials in this study: (**a**) PP/TiO_2_ 0.5 wt.%, (**b**) PP/TiO_2_ 2 wt.%, and (**c**) PP/TiO_2_ 4 wt.%.

The surface topology and roughness measurements conducted on the extruded filaments’ surface are shown in Figure 10. Namely, the PP/TiO_2_ 0.5 wt.% filament is presented in Figure 10a, while Figure 10b,c shows the surface topology and roughness of PP/TiO_2_ 2 wt.% and 4 wt.%, respectively.

The AFM analysis showed that the extruded filament surface was of an outstanding quality. In Figure 10, the worst scenario shows rather low roughness values of approximately 1 μm, which is almost 0.0006% of the filament diameter. Although the filaments produced for the purposes of this study had an overall smooth surface, the measurements (Figure 10) showed that the filler concentration had an effect on the filament roughness, as an increase in Rz was observed when moving from 0.5 wt.% (Figure 10a) to 4 wt.% (Figure 10c). The roughening effect is benchmarking the response to damping from the dynamic mechanical analysis, which implies agglomeration at high filler loadings.

#### 3.4.3. Melt Flow Volume Index (MVR) of Neat PP and PP/TiO_2_ Nanocomposites

**Figure 11:**
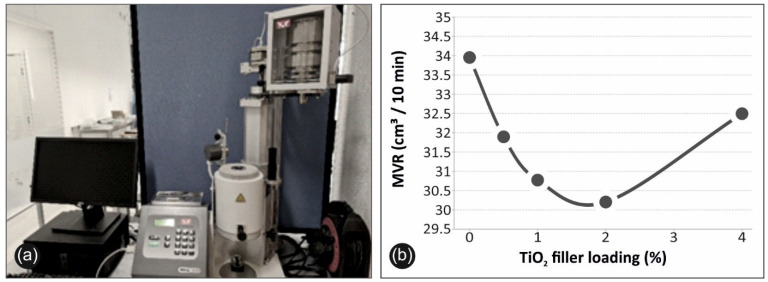
(**a**) Melt flow volume index (MVR) measurement setup, and (**b**) MVR values for the neat PP and PP/TiO_2_ nanocomposite at different filler loadings.

In this study, the MVR was determined (Figure 11) for the neat PP and the PP/TiO_2_ nanocomposites in order to perceive a clear view onto the thermoplastic materials processability, as well as to elaborate any potential processing problems that may be encountered during the 3D filamentous extrusion printing process. In other words, the MVR reflects the ease of flow of a melted thermoplastic polymer, thus providing a quality control index, which is quite important, especially for the FFF 3D printing manufacturing method. Figure 11a illustrates the experimental setup for the MVR measurements, while Figure 11b shows the MVR values for the neat PP and the PP/TiO_2_ nanocomposites at the different filler loadings.

As can be observed, MVR values with the increase of the filler concentration exhibited a constant decrease from ~34 cm^3^/10 min for the neat PP to ~30 cm^3^/10 min for the PP/TiO_2_ (2 wt.%), while for the case of the PP/TiO_2_ (4 wt.%) nanocomposite, the MVR value showed an increase to ~32.5 cm^3^/10 min, which was still lower, but close to the pure PP value. This drop in MVR values was expected, as the filler content was expected to increase the viscosity of the polymer melt, denoting the 2 wt.% filler loading as the level of maximum intermolecular interactions between the filler and/or TiO_2_/PP. However, as was indicated from DMA and AFM, potential filler agglomeration is likely to occur for the case of PP/TiO_2_ 4 wt.%, which affects the density change of the polymer melt or might be causing density inconsistencies. The high amount of filler is also likely to cause both a retarding effect on TiO_2_ on the PP crystals and the physical hindrance of the particles to the motion of the molecular chains. At the same time, the accumulated heat at high filler loadings is transferred to the surrounding polymer, causing potential chain cuts. All of these effects at PP/TiO_2_ 4 wt.% are likely to have a cumulative adverse effect on the viscosity, and correspondingly lead to an increase in MVR values, compared with the 0.5, 1, and 2 wt.% filler loadings [36,37,38].

## 4. Conclusions

**Figure 12:**
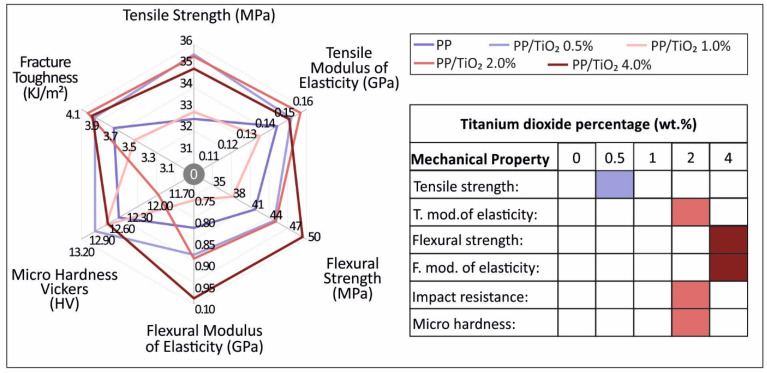
Summary of the mechanical properties for all of the materials tested in this study. The highest values measured for each test are marked as the filled percentage.

The study herein focused on the effect of TiO_2_ as a nanofiller in low weight-to-weight concentrations on the properties of the PP polymer material. Four nanocomposite types were fabricated and tested for the characterization of the mechanical, viscoelastic, physicochemical, and fractographic properties of the materials and for the evaluation of the nanofiller effect when compared with the pure polymer material. Figure 12 summarizes the mechanical properties of the pure PP and the nanocomposites fabricated in the current study. The highest values measured or calculated are marked on the right side of Figure 12. As was shown, TiO_2_ as a filler affects the mechanical properties of pure PP, with an increase observed in all of the cases studied. Specifically, the 0.5 wt.% sample showed a more improved tensile strength, while the 2 wt.% sample had the highest impact strength and microhardness values, and the 4 wt.% sample showed the highest strength in bending. Titanium dioxide seems to have no significant effect regarding the inherent structural characteristics of the composites. As was shown, the homogeneity of the material was kept constant under all of the filler percentages, while the increase of the filament roughness with the increase of the TiO_2_ concentration had little effect on the processability of the nanomaterials. Finally, filler ratio in the nanocomposite materials showed an effect regarding crystallinity; higher ratios increased the crystallinity existence in the nanocomposite materials.

Overall, the results of the current study showed a great potential for the PP/TiO_2_ nanocomposite to be used in AM applications. The nanomaterials were fabricated with an easy industrial ready thermomechanical process and their mechanical properties were improved when compared with the pure PP polymer, while the processability was not significantly affected, even at high loadings. As a future work, higher nanofiller concentrations could be prepared and tested, and further mixing possibilities with other nanoparticles could also be considered.

## Data Availability

The data presented in this study are available upon request from the corresponding author.
